# Powdery mildew-induced changes in phyllosphere microbial community dynamics of cucumber

**DOI:** 10.1093/femsec/fiae050

**Published:** 2024-04-10

**Authors:** Cong Yue, Changxia Du, Xiaodan Wang, Yinqing Tan, Xingchen Liu, Huaifu Fan

**Affiliations:** Collaborative Innovation Center for Efficient and Green Production of Agriculture in Mountainous Areas of Zhejiang Province, Key Laboratory of Quality and Safety Control for Subtropical Fruit and Vegetable, Ministry of Agriculture and Rural Affairs, College of Horticulture Science, Zhejiang A and F University, Hangzhou, Zhejiang 311300, China; Collaborative Innovation Center for Efficient and Green Production of Agriculture in Mountainous Areas of Zhejiang Province, Key Laboratory of Quality and Safety Control for Subtropical Fruit and Vegetable, Ministry of Agriculture and Rural Affairs, College of Horticulture Science, Zhejiang A and F University, Hangzhou, Zhejiang 311300, China; Collaborative Innovation Center for Efficient and Green Production of Agriculture in Mountainous Areas of Zhejiang Province, Key Laboratory of Quality and Safety Control for Subtropical Fruit and Vegetable, Ministry of Agriculture and Rural Affairs, College of Horticulture Science, Zhejiang A and F University, Hangzhou, Zhejiang 311300, China; Collaborative Innovation Center for Efficient and Green Production of Agriculture in Mountainous Areas of Zhejiang Province, Key Laboratory of Quality and Safety Control for Subtropical Fruit and Vegetable, Ministry of Agriculture and Rural Affairs, College of Horticulture Science, Zhejiang A and F University, Hangzhou, Zhejiang 311300, China; Collaborative Innovation Center for Efficient and Green Production of Agriculture in Mountainous Areas of Zhejiang Province, Key Laboratory of Quality and Safety Control for Subtropical Fruit and Vegetable, Ministry of Agriculture and Rural Affairs, College of Horticulture Science, Zhejiang A and F University, Hangzhou, Zhejiang 311300, China; Collaborative Innovation Center for Efficient and Green Production of Agriculture in Mountainous Areas of Zhejiang Province, Key Laboratory of Quality and Safety Control for Subtropical Fruit and Vegetable, Ministry of Agriculture and Rural Affairs, College of Horticulture Science, Zhejiang A and F University, Hangzhou, Zhejiang 311300, China

**Keywords:** co-occurrence network, cucumber powdery mildew, degree of disease, diversity, phyllosphere microbiota, relative abundance

## Abstract

As an important habitat for microorganisms, the phyllosphere has a great impact on plant growth and health, and changes in phyllosphere microorganisms are closely related to the occurrence of leaf diseases. However, there remains a limited understanding regarding alterations to the microbial community in the phyllosphere resulting from pathogen infections. Here, we analyzed and compared the differences in phyllosphere microorganisms of powdery mildew cucumber from three disease severity levels (0% < L1 < 30%, 30% ≤ L2 < 50%, L3 ≥ 50%, the number represents the lesion coverage rate of powdery mildew on leaves). There were significant differences in α diversity and community structure of phyllosphere communities under different disease levels. Disease severity altered the community structure of phyllosphere microorganisms, *Rosenbergiella, Rickettsia*, and *Cladosporium* accounted for the largest proportion in the L1 disease grade, while *Bacillus, Pantoea, Kocuria*, and *Podosphaera* had the highest relative abundance in the L3 disease grade. The co-occurrence network analysis of the phyllosphere microbial community indicated that the phyllosphere bacterial community was most affected by the severity of disease. Our results suggested that with the development of cucumber powdery mildew, the symbiotic relationship between species was broken, and the entire bacterial community tended to compete.

## Teaser

Changes of phyllosphere microbial community under different incidence grades of cucumber powdery mildew.

## Introduction

Microbes can benefit plants by decomposing compounds that cannot be absorbed (Chaparro et al. 2012, Almario et al. 2017, Li et al. 2022a), and can also stimulate plant defense responses (Berendsen et al. 2012). The phyllosphere is a complex microbial habitat (Vorholt 2012), as with the rhizosphere, host species and genotypes, complex environmental conditions, and various biological and abiotic factors can affect the phyllosphere microbial community (Singh et al. 2018, Beilsmith et al. 2019, Schlechter et al. 2019, Li et al. 2021). Microorganism-plant interactions have a huge impact on plant health (Getzke et al. 2019). For example, *Bacillus subtilis* effectively controlled soybean root rot and improved root biomass, plant height, and chlorophyll content (Jia et al. 2021). *Pseudomonas, Sphingomonas*, and *Bacillus* species in the phyllosphere, alleviated the damage to plants caused by the pathogen (Tsavkelova et al. 2007, Innerebner et al. 2011). Members of the *Methylobacterium, Microbacterium*, and *Stenotrophomonas* could improve the host plant's growth and nutritional status by producing indoleacetic acid and fixing nitrogen (Madhaiyan et al. 2015, Schoenfelder and Fraser 2019). When challenged by pathogens, plants increased the abundance of specific bacteria or fungi through biosynthesis, transport, and secretion processes (Canarini et al. 2019, Liu et al. 2020), and also attracted beneficial microorganisms in response to disease stress (Carrion et al. 2019, Liu et al. 2021).

Cucumber is an important vegetable crop in the world, which is often affected by various diseases during growth and development. Powdery mildew, caused by *Golovinomy*-*cescichoracearum* or *Podosphaera xanthii* (Kuzuya et al. 2006, Perez-Garcia et al. 2009), is widely distributed and spreads rapidly, making it one of the most devastating diseases of cucumber plants (Fukino et al. 2013). Currently, chemical agents are widely used to control powdery mildew (Cerkauskas and Ferguson 2014). However, the widespread and long-term use of chemical agents has made pathogenic bacteria resistant and caused environmental pollution (Fondevilla and Rubiales 2012, Rubiales et al. 2015). Biological control has the advantages of strong persistence and eco-friendly, which meets the requirements of agricultural sustainable development (Tanaka et al. 2017, Rur et al. 2018). Exploring the changes in the phyllosphere microbial community throughout the development of cucumber powdery mildew is of great significance for the biological control of the disease.

Therefore, in this study, we investigated changes in the phyllosphere microbial communities at different grades of disease with the objectives of: (1) understanding the diversity and structural differences of phyllosphere microbial communities at different levels of disease incidence, (2) investigating the correlation between the severity of cucumber powdery mildew and changes in the phyllosphere microbial communities, and (3) providing a theoretical basis for biological control studies of powdery mildew.

## Materials and methods

### Pathogen identification

Conidia of powdery mildew were collected from cucumber (Jin You No. 1) leaves infected with powdery mildew and made into 2.5–5.0 × 10^5^ conidia/mL of spore suspension. The spore suspension was inoculated on healthy cucumber seedlings by spray method for multiple isolations and purifications. Then, the powdery mildew spores were placed on the glass slide with sterile forceps and observed using a 10 × 40x optical microscope.

DNA was extracted from cucumber powdery mildew pathogen and amplified with ITS1 (5′-TCCGTAGGTGAACCTGCGG-3′) and ITS4 (5′-TCCTCCGCTTATTGATATGC-3′) primers. PCR reactions were performed with the following program: 3 min at 95°C, followed by 35 cycles of 30 s at 95°C, 25 s at 56°C, 1 min at 72°C, and a final extension step of 5 min at 72°C. The PCR products were detected on 1% agarose gel electrophoresis and sent to Tsingke Biotechnology Co., Ltd. (Beijing, China) for Sanger sequencing. The gene sequence of powdery mildew strains was uploaded to the NCBI database and obtained accession No. OQ216740. Mega 7.0 software was used for multiple sequence alignment. Construct a phylogenetic tree using the neighbor connection method and repeat testing with 1000 bootstraps.

### Powdery mildew pathogen inoculation and sample collection

Cucumber seeds were sown in medium with a ratio of peat, vermiculite, and perlite of 3:1:1 and cultivated in a climate chamber with 60% relative humidity, day/night temperature 25/15°C, photoperiod 16/8 h, light/dark. After two euphylla times, they were transplanted into the greenhouse and inoculated with a certain concentration of spore suspension on cucumber leaves. Two weeks later, based on the proportion of cucumber powdery mildew lesions (0% < L1 < 30%, 30% ≤ L2 < 50%, L3 ≥ 50%) (Fig. S1), leaf samples were collected from the same location and size using a five-point sampling method. The classification method of morbidity levels used was in accordance with China's national standard (GB/T 17980.30–2000) (Luo et al. 2019). Each disease level has 5 replicates, and all the samples were transported to the laboratory at refrigerated conditions.

### DNA extraction and purification

The method of microbial isolation in the phyllosphere was based on an earlier study (Redford and Fierer 2009, Bodenhausen et al. 2013, Xie et al. 2015). That is, after weighing the leaves, immersed them in a 100 mL sterile conical flask, and added a certain volume of 0.1 M PBS with 0.01% Tween 80. Shaken the flask at a speed of 250 rpm at 28°C for 30 min, and then ultrasonic treatment for 10 min. The phyllosphere microbiome were harvested using a 0.22 µm filter. Then transfer to −80°C refrigerator for storage to subsequent DNA extraction. According to the manufacturer's plan, the MP FastDNA^®^ SPIN Kit for Soil (MP Biochemicals, Solon, OH, USA) was used to extract DNA from the leaves surface. Bacteria were amplified using the specific primers 799F (5′-AACMGGATTAGATACCCKG-3′) and 1193R (5′-ACGTCATCCCCACCTTCC-3′). The fungi were amplified using ITS1F (5′-CTTGGTCATTTAGAGGAAGTAA-3′) and ITS2R (5′-GCTGCGTTCTTCATCGATGC-3′). Finally, the data was analyzed on Majorbio Cloud Platform's online platform.

### Processing of sequencing data

Raw sequence data reads were stored as FASTQ files, and the FASTQ files were quality filtered and analyzed using FASTTP (V0.19.6) and FLASH (V1.2.11) software to obtain optimized sequences (Caporaso et al. 2010, Edgar et al. 2011). Cluster all sequences with 97% similarity using UPARSE (V7.0.1090) to generate operational classification units (OTUs) (Edgar 2013). The representative sequences of OTUs with 97% similarity were classified and analyzed using the RDP classification database and the UNITE database (V8.0), respectively (Abarenkov et al. 2010).

### Statistical analysis

To assess phyllosphere microorganism diversity and richness, alpha diversity indexes, including Shannon index, Simpson's index, Chao and ACE index, were analyzed by Mothur (V1.30.2). Principal coordinate analysis (PCoA) and Venn diagram analysis using the vegan package in R software (V.3.2.5) (Lozupone et al. 2006). Calculation of species numbers in multiple groups or samples and differences in species composition among samples or subgroups at different taxonomic levels using R software (V3.3.1). The Kruskal-Wallis test was used to investigate the abundance and composition of phyllosphere microbial communities at different taxonomic levels. Linear discriminant analysis Effect Size (LEfSe) was used to identify dominant species in phyllosphere microbial communities of cucumber powdery mildew based on linear discriminant analysis (LDA threshold above 4).

To evaluate the effect of disease severity on the phyllosphere microbial community, the co-occurrence network of OTUs with the top 300 relative abundance was constructed. The “psych” package in the R language was used for pairwise comparison and Spearman correlation coefficient analysis. The correlation with the Spearman correlation coefficient (*R* > 0.6 or <–0.6) and statistical significance (*P* < 0.05) was selected for network construction. The interactive platform Gephi 0.9.2 was used to visualize the network. The node color represents the corresponding phylum for each OTU, the node size is proportional to the number of connections, and the edge represents the correlation among the nodes in the microbiome network.

## Result

### Pathogen identification

**Figure 1.**
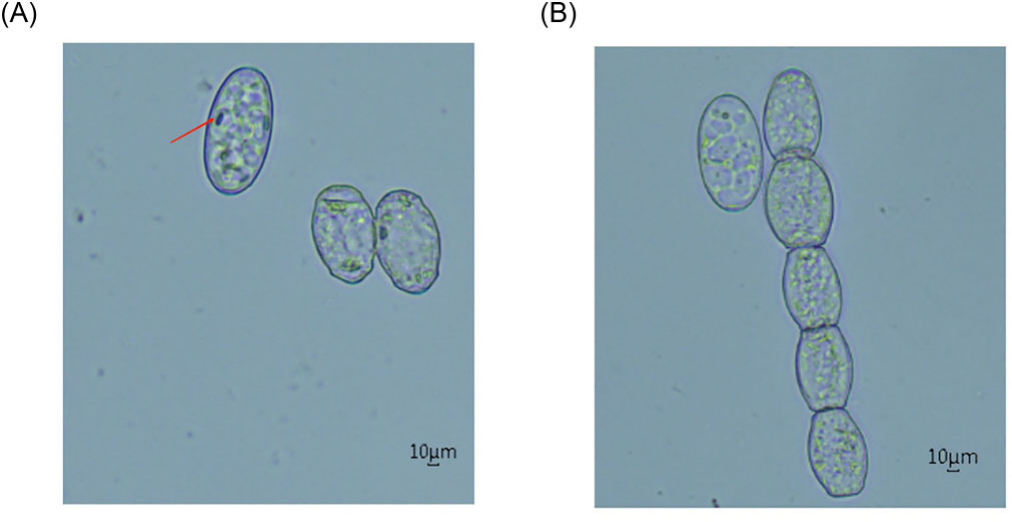
Observations of the cucumber powdery mildew pathogen by optical microscope (10 × 40x). (A) Conidia, Arrows point to fibrous bodies. (B) Conidiophores. Scale Bar: 10 µm.

As shown in Fig. 1, the conidia of powdery mildew pathogen were oval with fibrous structure, and the number of fibrous bodies in each conidia varies from 1 to 10. The conidiophores consisted of spores in tandem. Molecular biological identification confirmed that the ITS sequence of powdery mildew pathogen was highly consistent with that of *Podosphaera xanthii* (Fig. S2). Therefore, we determined that the pathogen causing cucumber powdery mildew was *Podosphaera xanthii* in the present study.

### Effect of disease severity on the diversity of microbial community in the phyllosphere

A total of 3600945 bacterial and 3857627 fungal raw sequences were obtained from the 15 leaf samples (Table 1). These original sequences were classified as 3104 bacterial and 1454 fungal OTUs with 97% similarity, respectively (Table S1). The rarefaction curves tend to be level off, meaning all samples reach sequencing depths (Fig. S3).

**Table 1.** Optimize sequence information.

Amplified Region	Samples	Sequences	Bases (bp)	Average Length	Average sequence bar
799F_1193R	15	3600945	1360774562	377	240063
ITS1F_ITS2R	15	3857627	925866375	240	257175

**Figure 2.**
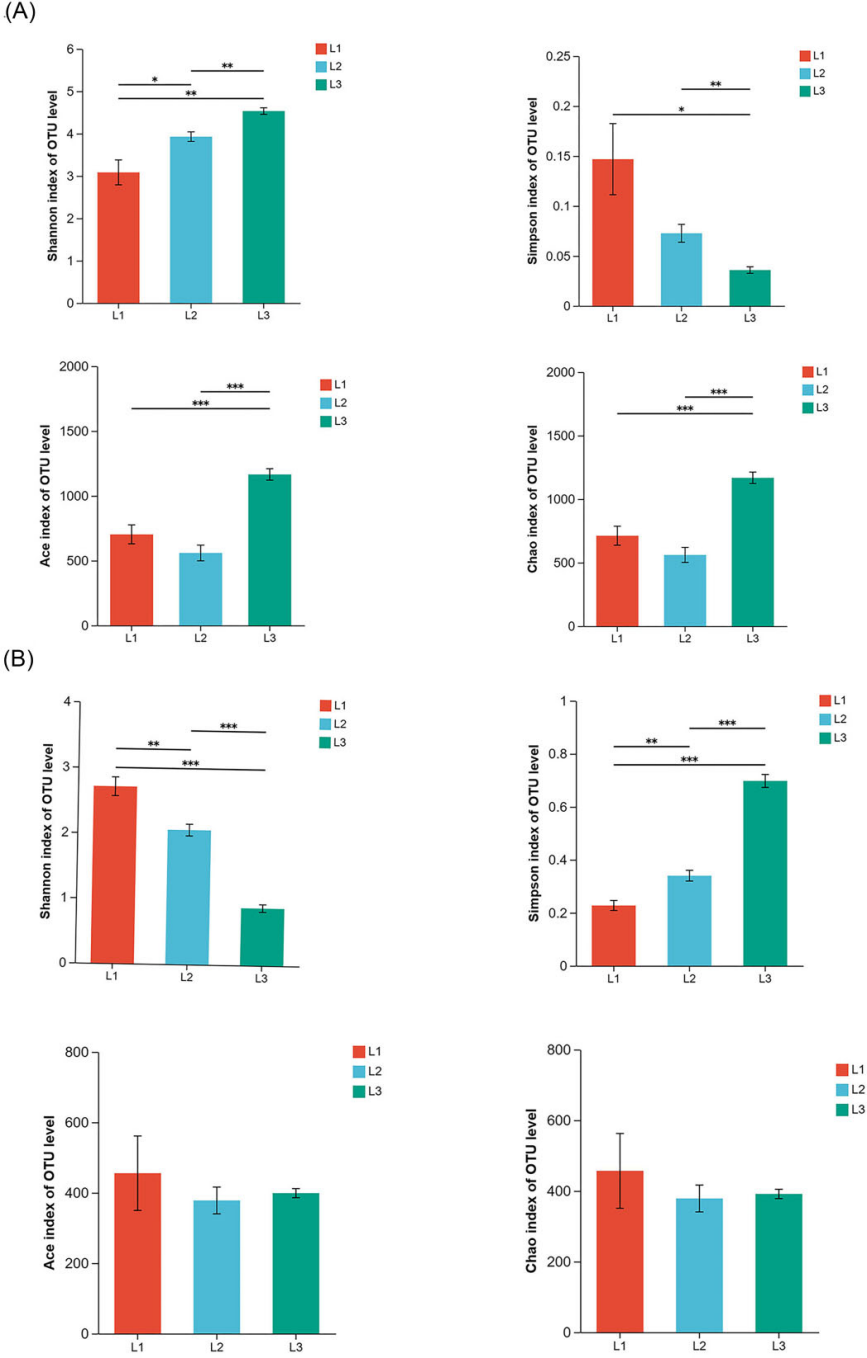
α diversity analysis in different degrees of disease. The α diversity indices including Shannon, Simpson indice, ACE and Chao in bacteria (A) and fungi (B) (Levels of significance are indicated as follows: *P* < 0.05 marked as *, *P* < 0.01 marked as ** and *P* < 0.001 marked as ***).

**Figure 3.**
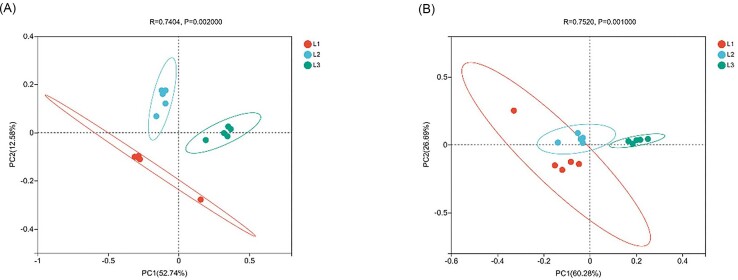
PCoA under different degrees of disease. (A) Bacteria. (B) Fungi.

The α diversity including Shannon, Simpson, Chao, and ACE indices. For bacterial communities α diversity, the diversity and richness of L3 incidence levels are significantly higher than other incidence levels (Fig. 2A). The fungal α diversity showed a decreasing trend with increasing disease grade, with no significant change in richness (Fig. 2B). The analysis of PCoA displayed that the phyllosphere bacterial community structure differed significantly at different incidence levels, while the fungal community structure differed less (*P* < 0.05) (Fig. 3).

### Effect of different disease severities on the composition and structure of the phyllosphere microorganisms

**Figure 4.**
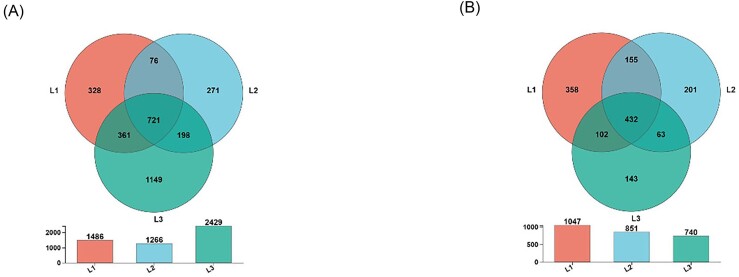
Phyllosphere microbial communities had unique or shared OTUs Venn diagrams at different disease levels. (A) Bacteria. (B) Fungi.

Venn diagrams were used to represent unique and shared OTU numbers at different incidence levels (Fig. 4). The highest number of unique OTUs was found in bacterial L3 (1149) and fungal L1 (358). The quantity of shared OTUs was 13.92% in bacteria and 16.38% in fungi.

**Figure 5.**
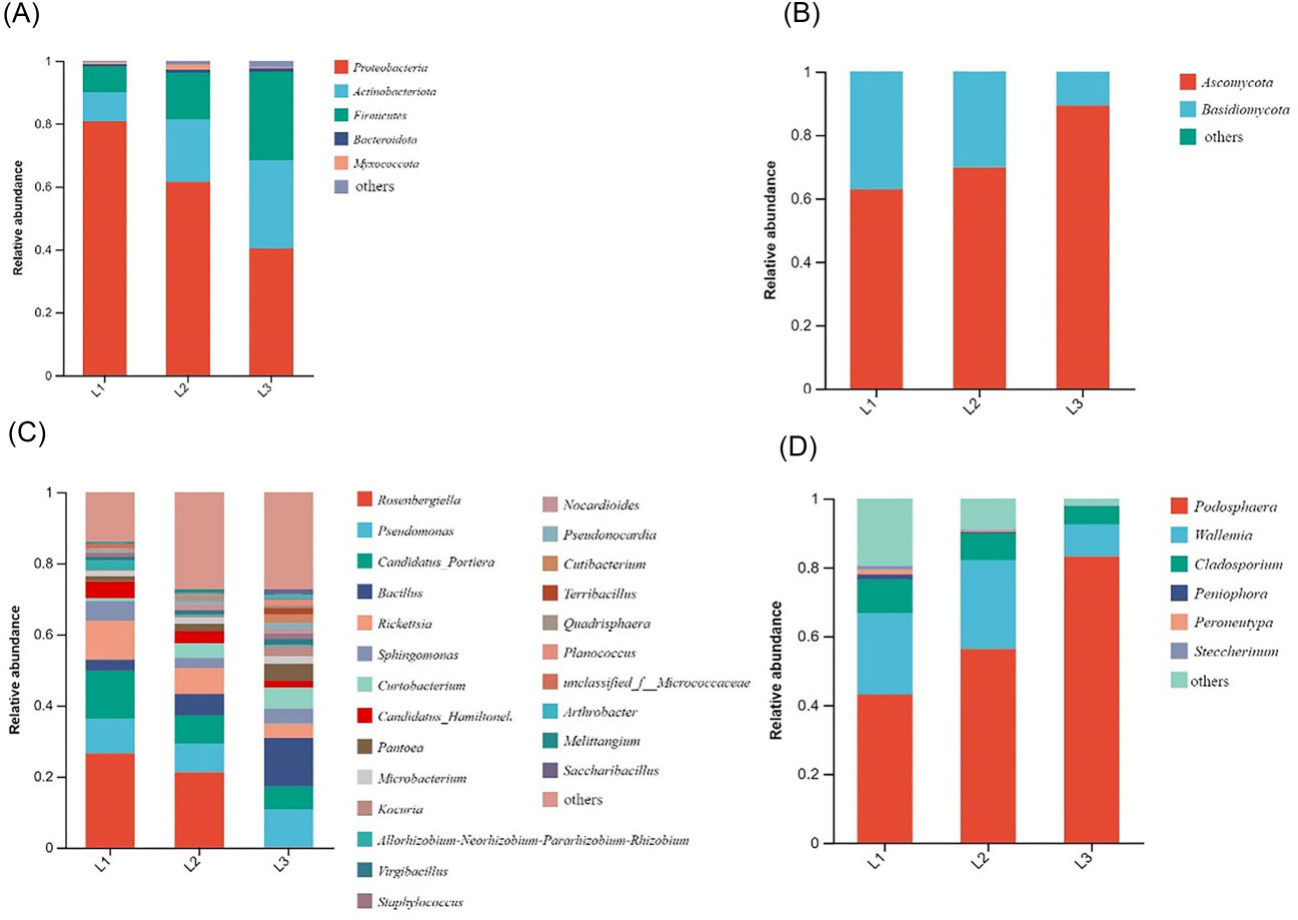
The relative abundance of bacteria (A and C) and fungi (B and D) at phylum and genera level under different incidence levels.

In the phyllosphere microbial community, we identified 32 phyla, 89 classes, 236 orders, 409 families, and 847 genera in bacteria communities (Fig. 5A). Proteobacteria, Actinobacteria, Firmicutes, and Bacteroidetes were the dominant bacterial phyla, accounting for 97.92%. Proteobacteria have the highest relative abundance (80.79%) in L1 grades. Actinobacteria and Firmicutes have the highest relative abundance (28.05% and 28.21%) in L3 grades. The fungal community identified 8 phyla, 29 classes, 85 orders, 242 families, and 529 genera (Fig. 5B). The dominant phyla were Basidiomycota and Ascomycota, accounting for 99.9%. The relative abundance of Ascomycota increased with the increasing severity of disease, accounting for 62.88%, 69.81%, and 89.34%, respectively. The relative abundance of Basidiomycota was highest in L1 (37.02%) and lowest in L3 (10.65%).

At the genus levels (Fig. 5C, D), the *Rosenbergiella* relative abundance in the bacterial community accounted for the largest proportion in L1 and nearly none in L3. Relative abundance of *Pseudomonas* showed a first increasing and then decreasing trend from L1 to L3. *Candidatus_Portiera* and *Rickettsia* relative abundance gradually declined and *Bacillus* gradually increased from L1 to L3. For the fungal community, *Podosphaera* was the most relative abundant in L3 (83.19%). *Wallemia* and *Cladosporium* were the most relative abundant in L1 (23.65% and 9.78%) in the fungal community.

### Analysis on the dominant species of the phyllosphere microbial community under different degrees of disease

**Figure 6.**
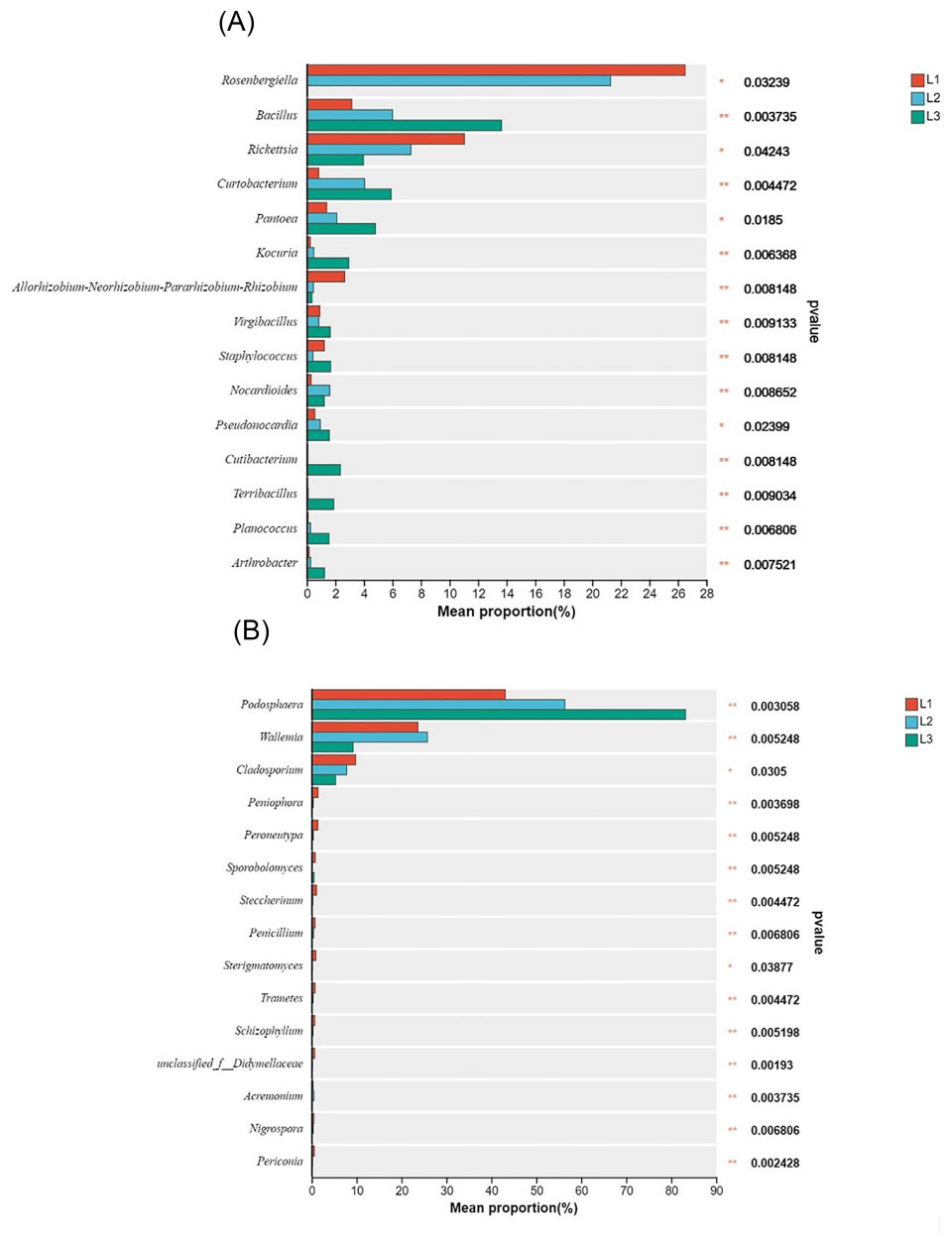
Analysis of differences in dominant genera under different disease levels. (A) Bacteria. (B) Fungi.

As shown in Fig. 6, there were significant differences between dominant genera of phyllosphere microbial communities at different disease levels. In the bacterial community, the relative abundance of *Rosenbergiella* and *Rickettsia* in L1 (26.5% and 11.04%) was higher than in L2 (21.28% and 7.29%) and L3 (0.07% and 3.98%). In contrast, the relative abundance of *Bacillus, Curtobacterium, Pantoea, Kocuria*, and *Virgibacillus* was the highest in L3. In the fungal community, the relative abundance of *Podosphaera* was highest in L3 (83.19%) and lowest in L1 (43.07%), *Wallemia* had the highest relative abundance in L2 (25.75%), *Cladosporium* was the highest in L1 and the lowest in L3.

**Figure 7.**
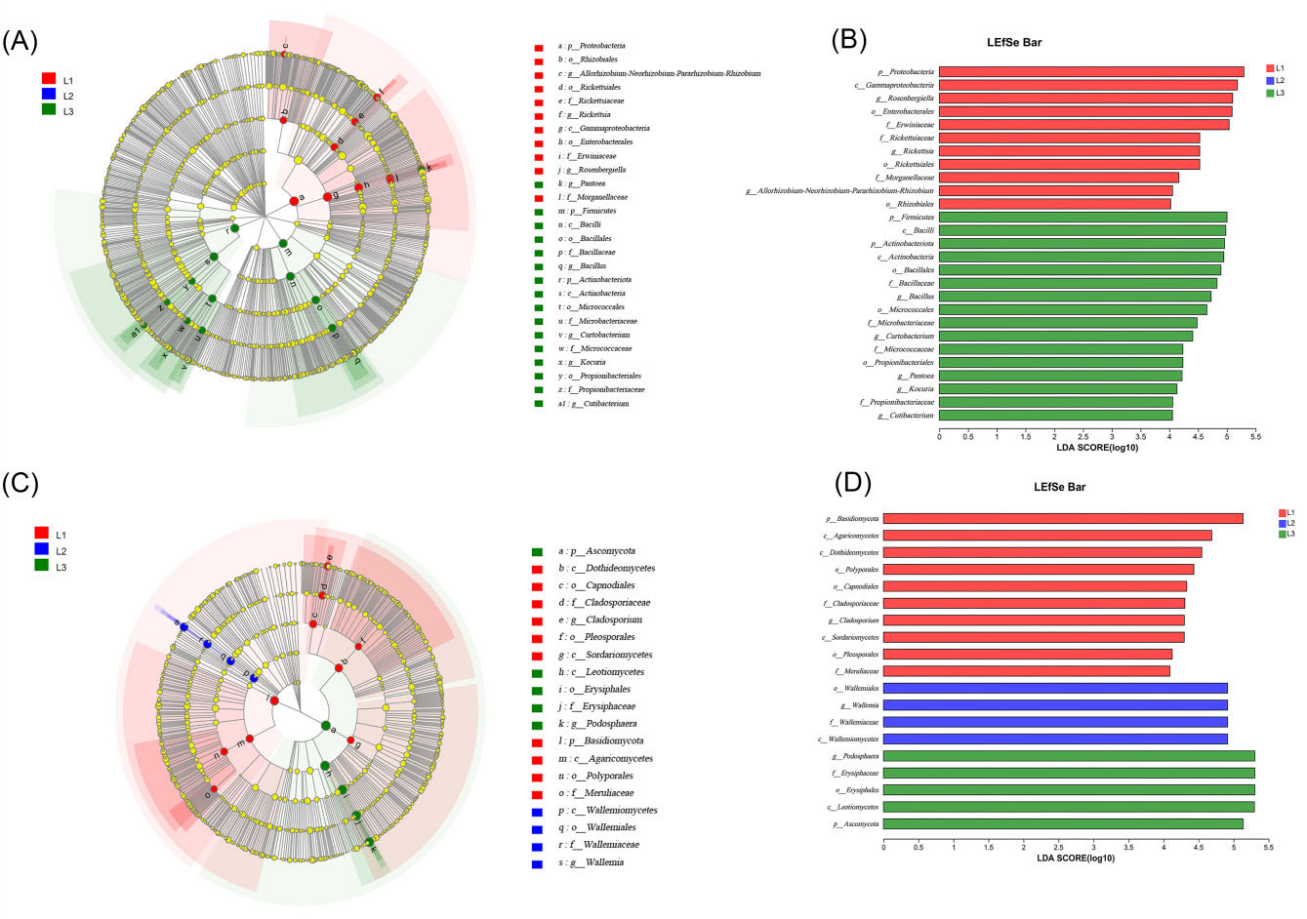
Discriminant analysis of species differences in phyllosphere community composition of cucumber powdery mildew under different degrees of incidence. The taxon from the inside to the outside of the branching diagram represents phyla, class, order, family, and genus respectively. The red, green, and blue nodes represent significantly different taxonomic levels in the three incidence levels (LDA threshold of 4). The yellow node indicates that there is no significant difference in the different groups. (A, B) Bacteria (C, D) Fungi.

LEfSe analysis revealed differences in the phyllosphere microbial communities at different levels of incidence from phylum to genus (Fig. 7). Bacterial community analysis found no dominant species enrichment in L2 compared with other ranks. Proteobacteria, Rhizobiales (from order to genus), Rickettsiales (from order to genus), Gammaproteobacteria (from order to genus), and Morganellaceae were significantly enriched in L1. In the L3 grade, Firmicutes and Actinobacteriota (all two from phylum to genus), Pantoea, Micrococcaceae (from family to genus) and Propionibacteriales (from order to genus) were enriched. The fungal communities Dothideomycetes (from class to genus), Pleosporales, Sordariomycetes, and Basidiomycota (from phylum to family) were enriched in L1. Wallemiomycetes (from order to genus) were enriched at L2. Ascomycota (from phylum to genus) was enriched in L3 grade.

### Analysis of microbial co-occurrence networks in the phyllosphere

**Figure 8.**
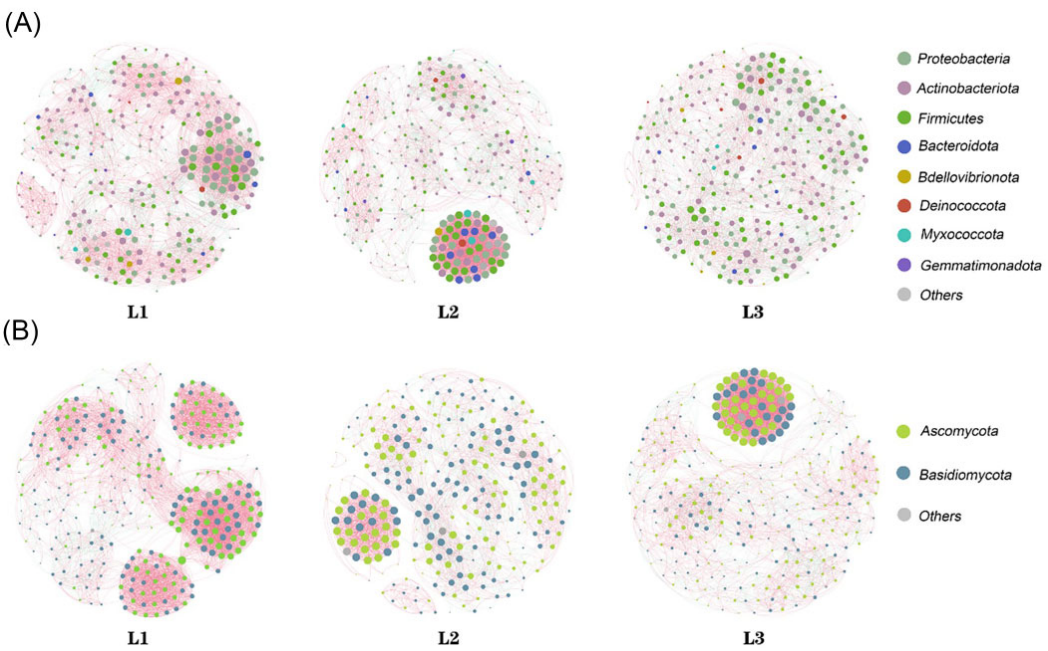
The co-occurrence network of OTUs with the top 300 relative abundance of bacteria (A) and fungi (B) in the phyllosphere under different degrees of disease. The size of each node is proportional to the number of connections. The color of nodes indicates their classification at the gate level. The red line and the blue line represent strong positive linearity (*r* > 0.6) and strong negative linearity (*r* < − 0.6) and relationships, respectively.

By analyzing the co-occurrence network, the complexity of the relationship between the species of the phyllosphere microbial community at different disease levels was revealed (Fig. 8, Table 2). It was found that most of the nodes of the co-occurrence network at different incidence levels belonged to 8 bacterial phyla and 2 fungal phyla. The number of bacterial community edges was inversely proportional to the degree of disease, and the number of fungal edges first decreased and then increased. In the bacterial network, there was no significant change in the percentages of positive correlation edges from L1 to L2 level, but the percentages of positive correlation edges obviously decreased at L3 level. While in the fugal co-occurrence network, it showed a trend of first increasing and then decreasing from L1 to L3 level. In addition, the average degree and the number of edges of L1 in both bacterial and fungal co-occurrence networks were higher than those of L2 and L3.

**Table 2.** Topological indices of each network in Fig. 8.

	Bacteria			Fungi		
Network properties	L1	L2	L3	L1	L2	L3
Number of nodes	299	299	299	299	267	299
Number of edges	4515	3984	3188	4873	2534	3614
The percentage of positive edges	76.01	77.21	51.29	93.78	71.51	81.35
The percentage of negative edges	23.99	22.79	48.71	6.22	28.49	18.65
Network diameter	8	7	6	7	7	7
Average path length	3.263	3.302	3.345	3.393	3.189	3.303
Average degree	30.201	26.649	21.324	32.595	18.981	24.174

## Discussion

Microbial communities have long been considered an important component of the plant ecosystem and contribute significantly to plant growth and disease resistance (Cordovez et al. 2019). An increasing number of studies have shown that when plants are infested by pathogens, plant microbial communities change and recruit beneficial microbes from the environment to cope with the effects of disease stress (Trivedi et al. 2012, de Assis Costa et al. 2018, Masenya et al. 2021). This phenomenon in which plants actively seek to cooperate with microorganisms to enhance stress resistance is known as the “call for help” strategy (Bakker et al. 2018). At present, there are many reports about the effects of pathogen on rhizosphere microbial communities, while the phyllosphere microbiome has been less studied (Yin et al. 2021).

The study found that the bacterial diversity and richness index at L3 level was significantly higher than that at L1 level. We speculated that with the increase of disease index, plants would recruit more beneficial bacteria to cope with disease stress. This is consistent with the research on cucumber angular leaf-spot (Luo et al. 2019). Conversely, fungal α diversity index decreased gradually with the increase of disease severity. PCoA analysis also showed that there were significant differences in microbial community structure in the phyllosphere at different disease severity. When powdery mildew occurred, *Podospaera xanthii* dominated in the phyllosphere microbiome, inhibiting the growth and reproduction of other fungi. This was the result of symbiosis and competition among microbial species. More and more powdery mildew pathogen was colonizing the leaves, seriously disrupting the original balance between phyllosphere microbiota, and plants exhibited more severe disease symptoms. On the other hand the *Podospaera xanthii* belongs to specialized parasitic fungi, which compete for host nutrition and inhibit host defense responses (Zhang et al. 2018). Therefore, with the increase of leaf powdery mildew coverage, cucumber leaves wither, become brittle, shrink, and lose photosynthetic function. Plants can maintain the stability of microbial communities by selecting and enriching certain microorganisms, which have achieved the effect of reducing diseases (Santhanam et al. 2015). At the phylum level, the bacterial community is mainly composed of Proteobacteria, Actinobacteria, Firmicutes, and Bacteroidetes in our research, and the fungal community is composed of Ascomycota and Basidiomycota, which are also the major components of the phyllosphere communities of most plants. The relative abundance of Actinobacteria and Firmicutes were highest at L3 grade. Actinobacteria and Firmicutes, which have the functions of producing high-grade secondary metabolites and participating in catabolic and transformation processes (Kim et al. 2011, Palaniyandi et al. 2013), are able to suppress fungal disease (Kim et al. 2007, Mendes et al. 2011). At the genus level, the relative abundance of *Rosenbergiella* and *Rickettsia* in the bacterial community was significantly negatively correlated with the disease grade. There is limited research on *Rosenbergiella* and *Rickettsia*, and their functions are still unclear. In contrast, *Bacillus, Pantoea*, and *Kocuria* were positively correlated with disease severity. *Pantoea* and *Bacillus* as biocontrol agents prevented plant diseases by antagonizing pathogens (Pusey 2002) and inducing plant defense responses (Johnson and Stockwell 1998, Wang et al. 2017, Fira et al. 2018). In addition, *Pantoea* has a unique biodegradation capability and can degrade herbicides and other toxic compounds (Giddens et al. 2002, Smits et al. 2010). The relative abundance of Wallemia and Cladosporium gradually decreases with the severity of powdery mildew, as they are pathogen of many plants (Abdelfattah et al. 2015).

Interactions between microorganisms are influenced by various biotic and abiotic factors (Faust and Raes 2012). Network analysis is a common method to study the correlation between different microbiomes (Li et al. 2017, Xu et al. 2020, Yuan et al. 2021). Therefore, the co-occurrence network was used to further explore the relationship among microbial populations and link microbial interactions with disease stress. The phyla with the higher relative abundance in the microbial networks were Proteobacteria, Actinobacteria, Basidiomycota, and Ascomycota. Proteobacteria and Ascomycota are the main microbial phyla, which play an important function in the co-occurrence network. They can rapidly decompose and absorb organic compounds and are more competitive than other fungi (Cui et al. 2019, Qiu et al. 2020). The network's topological features can indicate the complexity of microbial networks (Li et al. 2022b). Compared with L2 and L3, the L1 bacterial network showed a higher number of edge connections (4515), an average degree (30.201), and a lower average path length (3.263). These topological features showed that L1 has a more stable and complex ecological network, consistent with previous findings (Zhang et al. 2018). The high negative correlation index in co-occurrence networks is associated with ecological imbalances and competition between microorganisms (Gauthier et al. 2019), while the high positive correlation index indicates that there were less competitive interactions and more mutual benefits (Zhang et al. 2022). The number of edge connections, the proportion of positive correlations, and the average degree of fungal network showed first a downward and then increased trend, while the bacterial network showed a downward trend from L2 to L3. This indicated that the severity of the disease increased and the fungal network tended to be stable, while the bacterial network tended to be unstable. Together, these studies suggest that disease severity has a greater impact on bacterial communities.

In conclusion, in this study, it was found that disease severity was an important factor in the changes of the phyllosphere microbial community. There were significant differences in the structure of the phyllosphere microbial community under different disease grades. *Bacillus, Pantoea, Kocuria*, and *Podosphaera* were significantly enriched in the phyllosphere with the most severe disease. With the increase of the disease grade, the stability of the microbial community was broken, and the bacterial community tended to compete with each other rather than mutualism. These results provide a theoretical basis for exploring the changes of the phyllosphere microbial community of cucumber, screening and using beneficial microorganisms to inhibit the occurrence of cucumber powdery mildew. To further validate the correlation between disease severity and phyllosphere microbiota, a combination of culturomics and in vitro/in planta antagonistic activity assays is needed in future research.

## Supplementary Material

fiae050_Supplemental_File

## Data Availability

The raw sequences data of phyllosphere microorganisms at different disease levels of powdery mildew have been stored in the NCBI database under accession number: PRJNA932557.
