# Supplementary material for: Powdery mildew-induced changes in phyllosphere microbial community dynamics of cucumber
Source: FEMS Microbiol Ecol. 2024 Apr 10;100(5):fiae050. doi: 10.1093/femsec/fiae050 (PMC11062426; doi:10.1093/femsec/fiae050)
Supplement: fiae050_Supplemental_File [file fiae050_supplemental_file.docx]

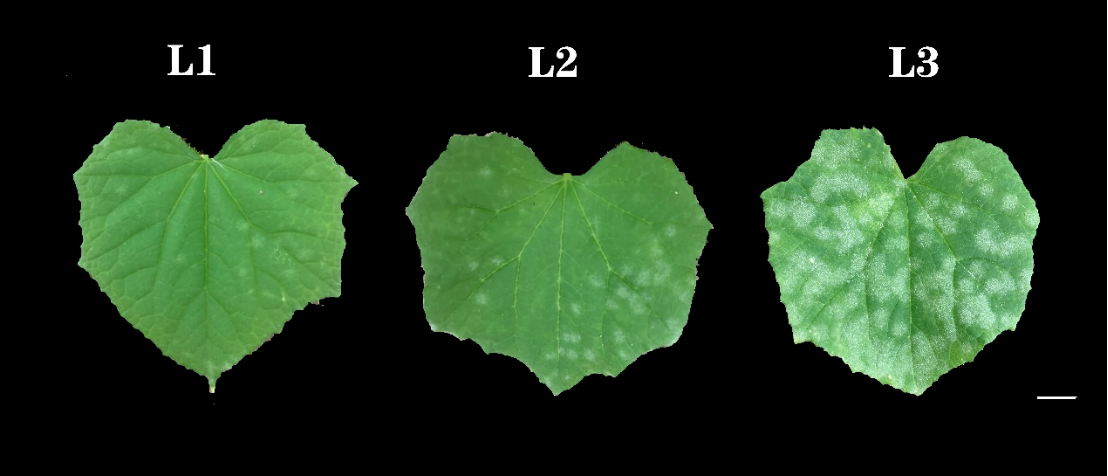


**Figure S1.** Cucumber leaves of different disease grades. 0% < L1 < 30%, 30% ≤ L2 < 50%, L3 ≥ 50% Scaleplate: 1 cm


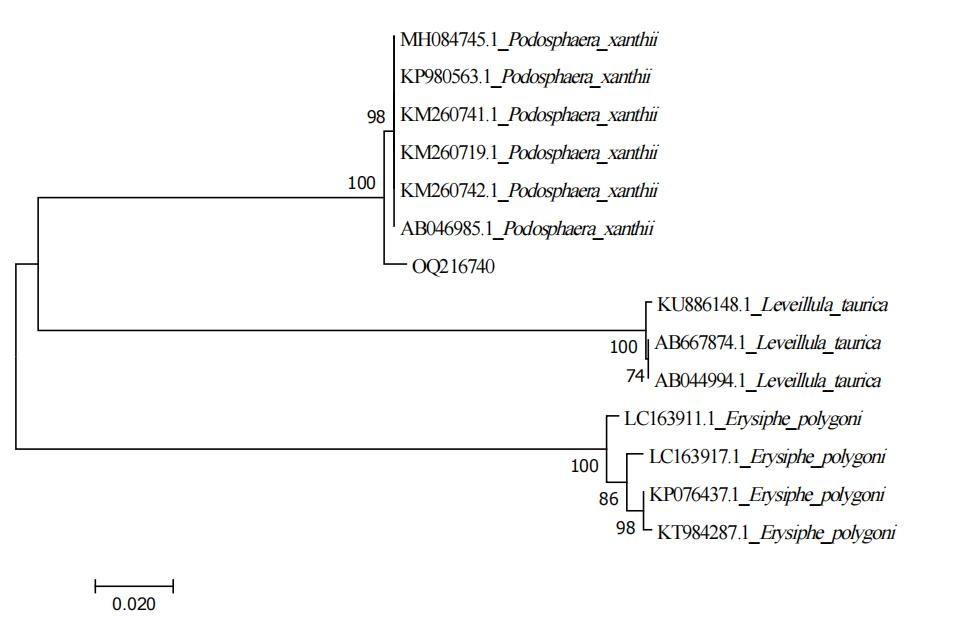


**Figure S2.** Phylogenetic tree based on ITS rDNA sequence analysis. OQ216740: Accession Number of cucumber powdery mildew pathogen


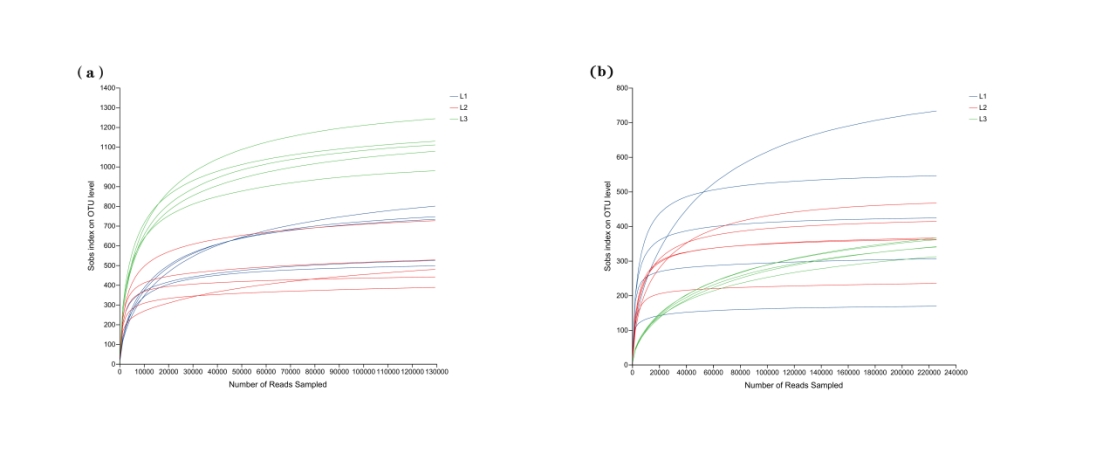


A

B

**Figure S3.** The Rarefaction curves for samples. (A) Bacteria, (B) Fungi

**Table S1.** Statistics of sequencing information

| Sample | Bacteria | | Fungal | |
| --- | --- | --- | --- | --- |
|  | Seq_ number | OTUs number | Seq_ number | OTUs number |
| L1_1 | 235243 | 766 | 231651 | 424 |
| L1_2 | 241805 | 517 | 229596 | 306 |
| L1_3 | 253207 | 785 | 228567 | 169 |
| L1_4 | 150365 | 524 | 238916 | 547 |
| L1_5 | 329538 | 895 | 283094 | 753 |
| L2_1 | 189536 | 514 | 285343 | 238 |
| L2_2 | 214062 | 541 | 264443 | 368 |
| L2_3 | 236736 | 456 | 248487 | 415 |
| L2_4 | 214204 | 402 | 300387 | 365 |
| L2_5 | 264162 | 750 | 226256 | 467 |
| L3_1 | 257601 | 1285 | 286478 | 383 |
| L3_2 | 271244 | 1118 | 240101 | 341 |
| L3_3 | 262081 | 1176 | 260323 | 351 |
| L3_4 | 262097 | 1021 | 255578 | 321 |
| L3_5 | 219064 | 1134 | 278407 | 369 |
| Total | 3600945 | 3104 | 3857627 | 1454 |
